# Successful treatment of lung adenocarcinoma complicated with a rare compound *EGFR* mutation L833V/H835L using aumolertinib: a case report and literature review

**DOI:** 10.3389/fphar.2023.1257592

**Published:** 2023-08-29

**Authors:** Linlin Li, Siyuan Huang, Liying Qin, Ningning Yan, Shujing Shen, Xingya Li

**Affiliations:** ^1^ Department of Medical Oncology, The First Affiliated Hospital of Zhengzhou University, Zhengzhou, China; ^2^ Academy of Medical Sciences, Zhengzhou University, Zhengzhou, China; ^3^ Department of Radiotherapy, The First Affiliated Hospital of Zhengzhou University, Zhengzhou, China

**Keywords:** NSCLC, epidermal growth factor receptor, L833V/H835L, aumolertinib, case report

## Abstract

**Background:** The deletion of exon 19 and the Leu858Arg mutation of exon 21 are the most frequently observed mutations in the epidermal growth factor receptor (*EGFR*) gene, and patients with these mutations have shown significant benefits from EGFR-tyrosine kinase inhibitors (TKIs). However, there exists a small subgroup of patients with uncommon/rare mutations of *EGFR*, including compound mutations, which display a high degree of heterogeneity in terms of clinical features and variable sensitivities to EGFR-TKIs. The understanding of these uncommon mutations and their response to targeted therapy is still unclear and requires further investigation.

**Case presentation:** We presented a case of a never-smoking patient with lung adenocarcinoma and brain metastasis. Initially, she received chemotherapy plus immune checkpoint inhibitor as first-line therapy as no *EGFR* mutations were detected by amplification-refractory mutation system-polymerase chain reaction. However, disease progressed rapidly. Subsequently, next-generation sequencing was carried out and revealed a rare compound mutation, L833V/H835L, in exon 21 of *EGFR*. As a result, she was switched to second-line therapy with the third-generation TKI aumolertinib, which demonstrated good efficacy. The patient was evaluated for a remarkable progression-free survival of 18 months and an overall survival of 29 months.

**Conclusion:** The present study supports that aumolertinib might be a good treatment option for advanced NSCLC patients with *EGFR* L833V/H835L mutation, particularly in patients with brain metastasis. Furthermore, conducting a comprehensive screening for gene mutations is crucial in effectively identifying potential oncogenic driver mutations and guiding mutation-targeted therapy decisions in clinical practice.

## Introduction

Lung cancer is a major contributor to cancer-related mortality globally, with non-small-cell lung cancer (NSCLC) comprising approximately 80%–85% of cases (Li et al., 2013). Targeted therapies aimed at oncogenic drivers have demonstrated greater efficacy when compared to standard chemotherapy for individuals with advanced NSCLC (Maemondo et al., 2010). Epidermal growth factor receptor (*EGFR*) mutations are prevalent in NSCLC, with a higher incidence of 40% in Asian patients and 20% in non-Asian patients (Liam et al., 2014). Among these mutations, the most common types are the in-frame deletion of exon 19 (19Del) and the Leu858Arg (L858R) mutation of exon 21, collectively known as common/classical mutations, accounting for approximately 85%–90% of EGFR gene mutations (Kobayashi and Mitsudomi, 2016; Galli et al., 2018; Khaddour et al., 2021). The remaining 10%–15% of *EGFR* mutations represent uncommon/rare mutations, which form a small and heterogeneous subgroup characterized by exhibiting distinct prognostic values and varied sensitivity profiles to targeted therapies (Harrison et al., 2020).

Compound mutations, comprising a small subset of uncommon *EGFR* mutations, have been reported to occur in approximately 4%–14% of all *EGFR* mutations (Yu et al., 2018; Chang et al., 2019). These compound mutations involve the presence of two or more mutational variants, which can include common mutations, uncommon mutations or both of them. Among compound mutations, the most frequent type involves the combination of an *EGFR* common mutation with a rare mutation (Passaro et al., 2021). The occurrence of two or more synchronous rare *EGFR* mutations, such as the L833V/H835L mutations, is exceedingly rare in lung cancer cases. Patients with compound mutations exhibit similar clinicopathologic features, including age, gender, smoking status, tumor sizes, and pathological stage, when compared to those with common or uncommon single *EGFR* mutations (Hayashi et al., 2020). The clinical outcomes of patients with compound mutations remain a subject of controversy, primarily due to the high heterogeneity characteristic of these mutations and the limited available data. Some researchers reported that patients with compound mutations had significantly poorer prognoses compared to those with common or single uncommon *EGFR* mutations (Kim et al., 2016). However, Antonio Passaro’s research demonstrated contrasting findings, indicating that patients with compound mutations exhibited similar responses and survival outcomes to those with common mutations. Specifically, when treated with EGFR-tyrosine kinase inhibitors (EGFR-TKIs), patients harboring compound mutations had a median progression-free survival time (mPFS) of 12.3 months and a median overall survival time (mOS) of 31 months. Notably, the mOS was longer in the compound mutations group (33.6 months) than in the single uncommon mutations group (12 months) (Passaro et al., 2019). The lack of standard treatments for compound mutations further contributes to the elusive nature of managing these cases.

Over the past two decades, significant progress has been made in genetic testing technology, leading to an improved understanding of disease biology and the mechanisms underlying tumor progression in NSCLC (Herbst et al., 2018). These advancements have facilitated the precise treatment of this condition. Currently, there are several commonly used techniques for detecting EGFR gene mutations, including direct sequencing (based on the Sanger sequencing method), amplification refractory mutation system (ARMS), and next-generation sequencing (NGS). Direct sequencing, although as a widely used technique, has some limitations, such as low sensitivity and being time-consuming and labor-intensive. Besides, ARMS utilizes appropriate primers designed with Taq DNA polymerase to detect specific known mutations. The amplified products are then analyzed using real-time fluorescence quantitative polymerase chain reaction (PCR). Compared to direct sequencing, ARMS offers higher sensitivity to gene mutations and is more convenient and rapid for clinical applications (Imyanitov et al., 2021). NGS, also known as high-throughput sequencing, represents a revolutionary advancement over traditional sequencing methods. NGS offers numerous advantages, including high throughput, high sensitivity, high specificity, and the ability to work with smaller patient sample sizes. However, it is important to note that NGS can be time-consuming in terms of data generation and analysis (Goodwin et al., 2021).

In this case study, a patient with lung adenocarcinoma and brain metastasis was initially assessed for EGFR gene mutations using ARMS-PCR, which failed to detect any positive mutations. Subsequently, NGS was employed, revealing the surprising identification of the L833V/H835L compound mutation of *EGFR*. The patient exhibited resistance to chemotherapy in combination with immune checkpoint inhibitor (ICI) treatment. However, a positive response was observed when the patient was treated with the third-generation TKI aumolertinib, leading to favorable PFS of 18 months. This case provides promising targeted therapy options for the L833V/H835L rare compound mutation of *EGFR,* and highlights the importance of conducting comprehensive gene analysis for precise treatment decisions.

## Case presentation

In July 2020, a 53-year-old Chinese female who had never smoked presented to our hospital with a 6-month history of cough and recent onset of dorsalgia. High-resolution computed tomography (HRCT) scans revealed a consolidative mass measuring approximately 2.6 × 1.8 cm in the right upper lobe (RUL) of the lung, as well as small nodules in the right middle lobe (RML). Magnetic resonance imaging (MRI) scans of the head showed metastases in the bilateral frontal and parietal lobes, cerebellar hemispheres, and left lateral ventricles. A CT-guided lung biopsy confirmed the presence of adenocarcinoma in the mass located in the RUL. Based on the TNM staging system outlined in the American Joint Committee on Cancer (AJCC) version 8.0, the patient was diagnosed with stage IVB adenocarcinoma NSCLC, with brain metastases (T4N0M1c). The patient’s performance status was classified as PS1. The ARMS-PCR was carried out for genomic analysis, while no mutations were detected in exons 18 and 21 of the EGFR gene.

**FIGURE 1:**
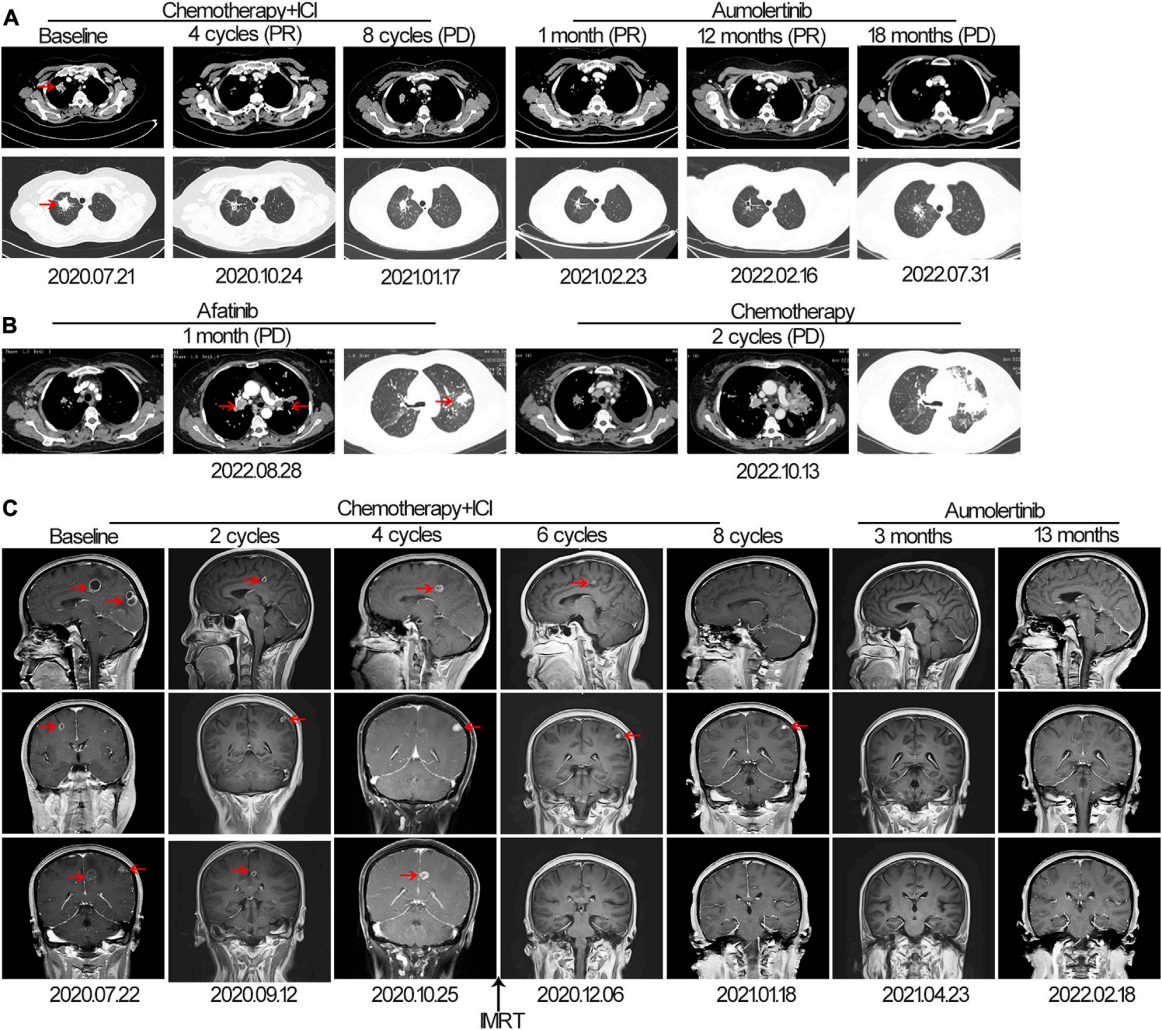
The tumor progression of the patient before and after treatment. **(A, B)** Representative lesions in the lung lobes according to computed tomography images at various points. **(C)** Magnetic resonance images at various points. The tumor is indicated by red arrows. ICI, immune checkpoint inhibitor; IMRT: intensity-modulated radiotherapy; PD, progressive disease; PR, partial response.

The patient was successfully enrolled in a study titled “A phase III, randomized, double-blind, multi-center study of AK105 combined with carboplatin plus pemetrexed *versus* placebo combined with carboplatin plus pemetrexed as first-line therapy in patients with metastatic non-squamous NSCLC” (AK105-301, ClinicalTrials.gov Identifier: NCT03866980). AK105 (also called penpulimab), a recombinant humanized anti-PD-1 monoclonal antibody, has been approved by the National Medical Products Administration (NMPA) of China for the treatment of advanced squamous NSCLC. Following four cycles of penpulimab combined with pemetrexed plus carboplatin treatment, a HRCT scan revealed a significant reduction in the size of nodules in the RUL of the lung (measuring 1.8 cm × 0.8 cm) compared to the baseline (Figure 1A). However, a head MRI showed an increase in the number and size of brain metastases compared to the baseline (Figure 1C). After a multidisciplinary team discussion, it was decided to continue the current treatment and administer additional radiotherapy for the brain metastases. The patient underwent intensity-modulated radiotherapy (IMRT) for the brain metastases, receiving a total dose of 45 Gy delivered in daily fractions of 4.5 Gy. Subsequent MRI scans demonstrated effective control of the brain metastases (Figure 1C). The patient continued treatment with additional four cycles of immunotherapy plus chemotherapy. However, a follow-up HRCT scan on 17 January 2021, revealed an increase in the size of the mass in the RUL compared to the scan performed on 24 October 2020. Due to disease progression, the patient discontinued participation in the clinical trial. The PFS for the first-line therapy was reported to be 5 months.

**FIGURE 2:**
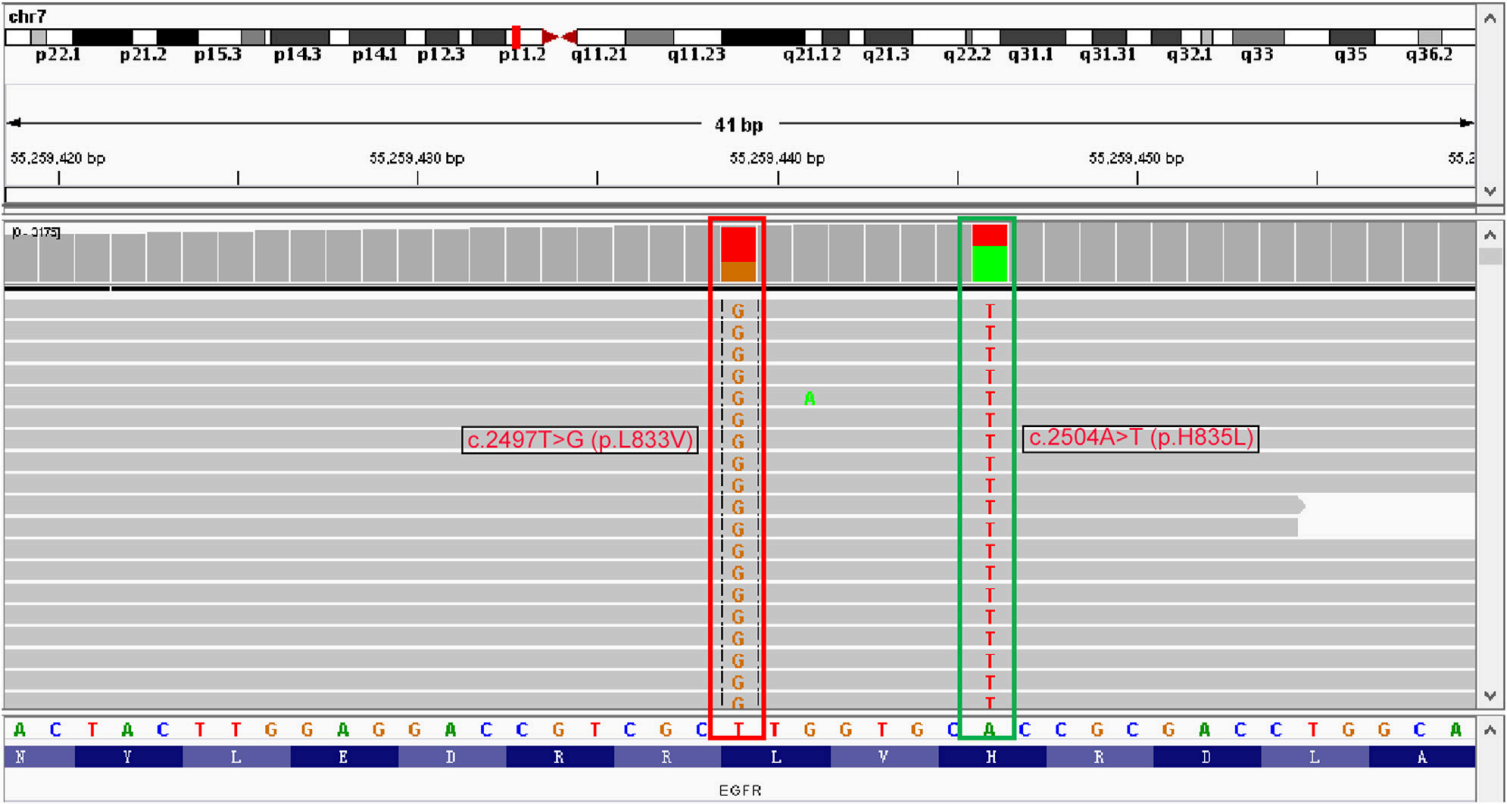
Identification of L833V and H835L in EGFR exon 21 by next-generation sequencing.

**FIGURE 3:**
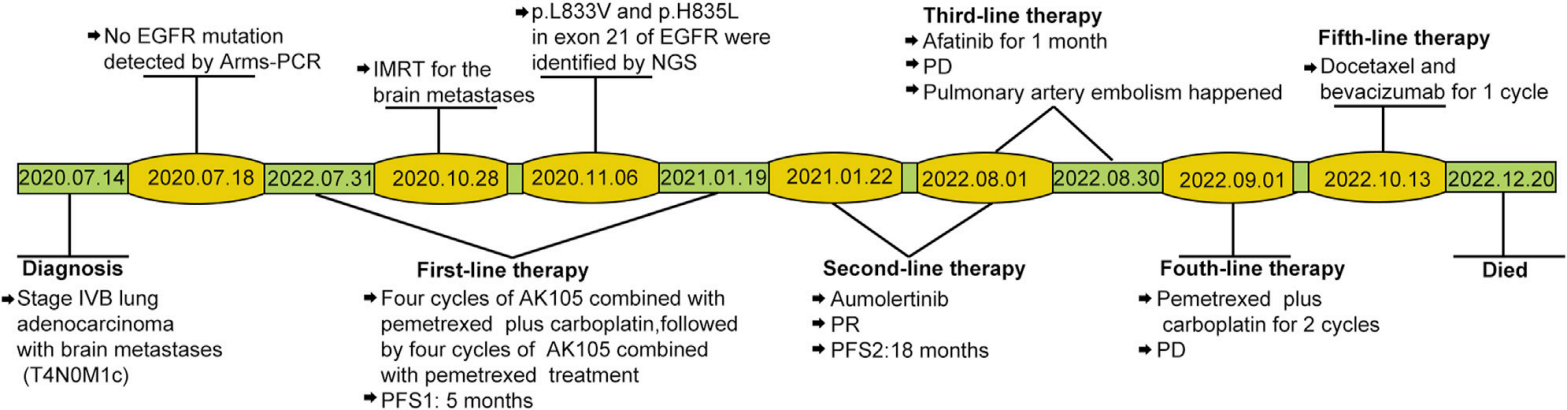
Case timeline. PFS, progression-free survival. IMRT: intensity-modulated radiotherapy; PD, progressive disease; PR, partial response.

For initially detecting the mutation status of *EGFR*, due to financial pressures, the patient chose to send tissues to the central laboratory for free ARMS-PCR detection rather than undergoing NGS at her own expense. Considering the patient’s non-smoking status and the limitations of the initial gene detection method, which was ARMS-PCR, known for its challenges in detecting rare mutations, a tumor tissue sample was subjected to the NGS analysis through a 425-gene panel (Accession ID: SAMN36750467, https://www.ncbi.nlm.nih.gov/biosample/36750467). This comprehensive analysis identified the presence of synchronous mutations, p.L833V and p.H835L, in exon 21 of the EGFR gene (Figure 2). Subsequently, the patient received aumolertinib, a third-generation EGFR-TKI, as second-line therapy starting from 22 January 2021. Follow-up chest HRCT and brain MRI scans conducted after aumolertinib treatment revealed a significant reduction in the size of the lung mass in the RUL (Figure 1A) and complete disappearance of the lesions in the occipital lobe (Figure 1C). These findings indicated a partial response (PR) to the treatment, with no significant adverse effects reported during this period. After 18 months of aumolertinib treatment, disease progression was observed in the HRCT scan conducted in 31 July 2022, with growth of the mass in the RUL and newly appeared nodules in both lobes compared with Apial, 2022. The patient then received afatinib as third-line therapy for 1 month, but it proved ineffective in controlling growth of the mass. Although the patient were switched to two cycles of pemetrexed plus carboplatin treatment, she began to troubled with dyspnea. The tumour continued growing aggressively in the bilateral lung lobes (Figure 1B). Subsequently, one cycle of docetaxel plus bevacizumab therapy was administrated, the tumor was still out of control. Unfortunately, she succumbed to respiratory failure on 20 December 2022. The OS duration for the patient was 29.0 months (Figure 3).

## Discussion

This case report, for the first time, presented a patient with rare compound *EGFR* mutations of L833V/H835L who benefited from aumolertinib for 18 months. The case report had several unusual features: the patient harbored rare compound *EGFR* mutations of L833V/H835L, which were neglected by ARMS-PCR, while were detected through NGS; the patient also had brain metastasis; responded well to the third-generation TKI aumolertinib, while showing resistance to the second-generation TKI afatinib; the patient primary resistance to platinum-based chemotherapy and immunotherapy was observed. While the ARMS method is known for its sensitivity and reliability in detecting specific mutations, it has limitations in identifying rare mutations (Ellison et al., 2010). In this case, the significance of NGS in identifying molecular alterations, particularly unknown or rare gene mutations, was highlighted (Fernandez-Marmiesse et al., 2018).

In the context of compound mutations, the occurrence of two or more synchronous rare *EGFR* mutations like L833V/H835L in lung cancer is extremely rare (Hayashi et al., 2020). Despite inconsistent conclusions regarding the prognoses of compound mutations, there are some evidence to suggest that the presence of two rare sensitive mutations, or combinations that include deletions of exon 19 or the L858R point mutation, may be associated with a more favorable response to EGFR-TKIs and a longer survival time when compared to single rare mutations (Rossi et al., 2022). The present study demonstrated that the patient with the L833V/H835L *EGFR* compound mutation achieved an OS of 29 months, which is comparable to the OS observed in patients with common EGFR mutations. These findings provide evidence that the L833V/H835L might be sensitive *EGFR* mutation and a good prognostic indicator, patients harboring the L833V/H835L mutation can have a good prognosis.

Cases of the L833V/H835L compound rare mutations were seldom reported, and their response to EGFR-TKIs remained largely unknown. To the best of our knowledge, only 18 patients with the L833V/H835L *EGFR* mutation had been reported at that time. Among these cases, 9 patients had been documented with clinical characteristics and therapeutic response, including the patient in our study (Table 1). The first case was reported in 2011, where they identified four patients harboring H835L/L833V mutations from a database of 1,090 NSCLC patients. Among them, an 89-year-old Asian man received first-generation TKI, gefitinib, as second-line therapy and experienced 8.5 months of disease stability without progression (Yang et al., 2011). In the early stage, gefitinib was frequently administered to treat patients with L833V/H835L mutations, this preference may be attributed to previous findings that gefitinib exhibit activity in patients with compound EGFR-mutations (the response rate was 50%), albeit with lower efficacy compared to patients with common mutations (Yu et al., 2018). Li et al. also proved a more favorable response to gefitinib in patients with compound *EGFR* mutations compared to those with a single rare variant through molecular dynamics simulations assay (Li et al., 2022). Among the 9 cases included in Table 1, 4 patients were treated with the gefitinib. Among them, 3 patients demonstrated a positive response to gefitinib, with PFS of 8.5 months, 15 months, and over 18 months, respectively. However, the fourth patient with brain metastasis showed primary resistance to gefitinib and experienced rapid progression after just 1 month of treatment, this exposes the disadvantage of gefitinib in control brain metastasis. The second-generation TKIs afatinib may be effective for uncommon mutations as showed in series of *post hoc* analysis (Russo et al., 2019). However, it is important to note that majority of the patients enrolled in these studies had mutations of G719X, S768I, or L861Q, few patients had compound mutation. In Table 1, 3 cases received afatinib treatment, and they exhibited moderate responses, with PFS of over 2 months, 7 months, and above 10 months, respectively, which showed no advance to gefitinib treatment.

**TABLE 1:** Reported cases of EGFR L833V/H835L mutation in NSCLC and response to therapy.

Author/Report year	Age(y)/Gender	Race	Smoker	CNS metastases	Mutations	Treatment	PFS (months)
Yang et al. (2011)	89/M	Asian	Yes	No	L833V/H835L	Gefitinib (second-line)	8.5
Zhuang et al. (2013)	48/M	Asian	No	No	L833V/H835L	NA	NA
Frega et al. (2016)	70/M	Non-Asian	Yes	No	L833V/H835L/E709K	Afatinib	>2
Qin et al. (2018)	36/M	Asian	No	Yes (brain parenchyma)	L833V/H835L/R670W	Gefitinib (first-line), Afatinib (third-line), Osimertinib (fifth-line)	1 (Gefitinib), 7 (Afatinib), >3 (Osimertinib)
Li et al. (2019)	77/F	Asian	No	No	L833V/H835L	Gefitinib	15
Long et al. (2020)	65/M	Asian	No	No	L833V/H835L	Afatinib	>10
Yang et al. (2022)	65/M	Asian	Yes	No	L833V/H835L	gefitinib	>18
Smith et al. (2023)	60/F	NA	Yes	Yes (leptomeninx)	L833V/H835L	Osimertinib	19

Abbreviations: AD, adenocarcinoma; F, female; M, male; NA, not available; CNS, central nervous system.

The effect of third-generation TKIs on patients with compound mutations is seldom known (Rossi et al., 2022). Since the approval of osimertinib as first-line therapy for *EGFR*-mutated advanced NSCLC by Food and Drug Administration, there has been a growing interest among researchers to explore the role of osimertinib in uncommon mutations (Soria et al., 2018). In a prospective phase II study (KCSG-LU15-09), 36 patients with uncommon *EGFR* mutations, excluding exon 20 insertions, demonstrated a favorable response to osimertinib (Cho et al., 2020). The study reported a 50% response rate and a mPFS of 8.2 months in these patients. In a retrospective study involving 60 patients with uncommon *EGFR* mutations, including 27 patients (45%) with compound *EGFR* mutations, the use of osimertinib as a first-line therapy showed promising results. The study reported a 61% objective response rate (ORR), a mPFS of 9.5 months, and a median OS of 24.5 months in these patients) (Bar et al., 2023). Furthermore, a clinical trial evaluating the efficacy of the third-generation TKI aumolertinib in advanced NSCLC patients with uncommon *EGFR* mutations was conducted in 2021 (NCT04785742).

In the case series of the 9 patients harboring the L833V/H835L mutation, 3 of them received third-generation TKIs, including osimertinib and aumolertinib. All 3 patients had developed brain parenchyma or leptomeningeal metastases. Remarkably, all of them exhibited a favorable response to the treatment, with no disease progression observed for 18 months, 19 months, and more than 3 months, respectively. One particularly interesting case involved a 36-year-old man who was a nonsmoker and had triple uncommon *EGFR* mutations: R670W in exon 17 and L833V/H835L in exon 21. Despite experiencing disease progression after six previous lines of therapy, including a 1-month course of gefitinib, chemotherapy, and afatinib, the patient demonstrated a positive response to osimertinib as the seventh-line therapy, with a duration of more than 3 months (Qin et al., 2018). The use of third-generation TKIs has shown superior efficacy in the treatment of central nervous system metastases (CNS) compared to platinum chemotherapy and first/second-generation EGFR-TKIs (Gen et al., 2022). This is attributed to their ability to cross the blood-brain barrier and effectively penetrate the CNS. Based on these findings, it can be concluded that the L833V/H835L compound mutation, which consists solely of rare mutations, exhibits a favorable response to third-generation EGFR-TKIs. Furthermore, third-generation TKIs have demonstrated superior effects compared to first-generation TKIs in patients with CNS metastases.

The mechanisms of action of the L833V/H835L mutation remains unknown. Actually, we did not find any reports of the individual missense mutation in either L833V or H835L by mining literature. The L833V mutation tends to co-occur with other *EGFR* mutations, most commonly with H835L, and the remaining, more rarely, with L858R (Kian et al., 2023), G719X (Zhou et al., 2022), or V744M (Pan et al., 2023). The co-mutations of L833V/H835L might cooperatively promote the oncogenic activation and fully phenotype transformation. Compared to osimertinib, aumolertinib undergoes structural modifications with the addition of a cyclopropyl group, which enhances its lipophilicity and facilitates better penetration through the blood-brain barrier. This unique property of aumolertinib makes it particularly effective in treating NSCLC patients with brain metastases (Talele, 2016; Garcia et al., 2021). In our case, since the patient had multiple brain metastases, aumolertinib was administered as second-line therapy and resulted in a favorable response for 18 months. This is the first reported case of a patient with the L833V/H835L mutation responding well to aumolertinib. Following resistance to aumolertinib, the patient was switched to afatinib and two lines of chemotherapy, however, the disease progressed rapidly. This suggests the possibility of secondary resistance mutations emerging, which confer resistance to afatinib. Our findings suggest that aumolertinib may be the optimal treatment option for patients with the *EGFR* compound mutation L833V/H835L and brain metastases.

## Conclusion

In this case report, the patient received aumolertinib as a second-line therapy achieving a PFS of 18.0 months, and an OS of 29.0 months. The occurrence, treatment and outcome of *EGFR* compound mutation L833V/H835L were seldom reported previously, this report is the first clinically significant case of aumolertinib in the treatment of this subtype of compound mutation, with encouraging results. Successful treatment with aumolertinib could provide a new treatment option for L833V/H835L *EGFR* mutation, especially for patients with CNS metastasis. However, data from case reports provided only some supportive evidence, further studies are required to validate the efficacy of third-generation TKIs in these patients. This report also emphasize the importance of utilizing complementary genetic analysis to ensure accurate detection.

## Data Availability

The datasets presented in this study can be found in online repositories. The names of the repository/repositories and accession number(s) can be found below: https://www.ncbi.nlm.nih.gov/sra/PRJNA999860.
